# Virtually Being Lenin Enhances Presence and Engagement in a Scene From the Russian Revolution

**DOI:** 10.3389/frobt.2018.00091

**Published:** 2018-08-23

**Authors:** Mel Slater, Xavi Navarro, Jose Valenzuela, Ramon Oliva, Alejandro Beacco, Jacob Thorn, Zillah Watson

**Affiliations:** ^1^Department of Computer Science, University College London, London, United Kingdom; ^2^Department of Clinical Psychology and Psychobiology, University of Barcelona, Barcelona, Spain; ^3^Virtual Bodyworks S.L., Barcelona, Spain; ^4^BBC Research and Development/BBC VR Hub, London, United Kingdom

**Keywords:** virtual reality, presence, body ownership, cultural heritage, history, Russian revolution, Lenin, immersive journalism

## Abstract

Virtual Reality (VR) has been widely applied to cultural heritage such as the reconstruction of ancient sites and artifacts. It has hardly been applied to the reprise of specific important moments in history. On the other hand immersive journalism does attempt to recreate current events in VR, but such applications typically give the viewer a disembodied non-participatory role in the scene of interest. Here we show how VR was used to reconstruct a specific historical event, where a famous photograph was brought to life, showing Lenin, the leader of the 1917 October Russian Revolution, giving a speech to Red Army recruits in Moscow 1920. We carried out a between groups experimental study with three conditions: Embodied—where the participant was first embodied as Lenin and then later in the audience watching Lenin; Included—where the participant was not embodied as Lenin but was embodied as part of the audience; and Observing—where the participant mainly viewed the scene from a disembodied third person point of view. Twenty participants were assigned to each of the three conditions in a between-groups design. We found that the level of presence was greatest in the Embodied and Included conditions, and that participants were least likely to later follow up information about the Russian Revolution in the Observing condition. Our conclusion is that if the VR setup allows for a period of embodiment as a character in the scenario then this should be employed in order to maximize the chance of participant presence and engagement with the story.

## Introduction

There has been an enormous amount of work on the application of virtual reality to cultural heritage, see the review by Remondino and Rizzi (2010) which reports several examples. Cultural heritage applications overwhelmingly concentrate on sites and objects, for example, a temple Sundstedt et al. (2004), Michelangelo's statue of David or items from Ancient Rome (Levoy et al., 2000), the Monastery of Santa Maria de Ripoll in Spain (Besora et al., 2008; Callieri et al., 2011), reconstruction of plaques and inscriptions of condemned people in Ancient Rome (Manferdini et al., 2016), even underwater European heritage (Bruno et al., 2017), and many other examples. There are also applications that attempt to recreate extended events—for example, the World War 2 D-Day Normandy landings represented in a video game (Rejack, 2007), or a simulation of the world's first city where participants can navigate through the environment and interact with virtual inhabitants (Ijaz et al., 2017). Rizvic (2014) shows how many cultural heritage applications are story guided, and Allison (2008) reviews such applications in the context of history education.

There has been little work, however, on the reconstruction of specific moments in time, such as a speech by a famous leader. Here we concentrate on immersing people using virtual reality (VR) in the partial reconstruction of an event rather than an experience of being in a simulated extended event. Moreover, unlike a game where the user has options, here we were interested in participants living through an event in something like the way it happened. At the time of writing it is the centenary of the 1917 October/November Revolution in Russia. We were interested in how the portrayal of an event following the Revolution might give people the illusion of having been there and taken part in it, and also how to engender sufficient interest so that they would later follow-up on those events after their exposure.

**Figure 1:**
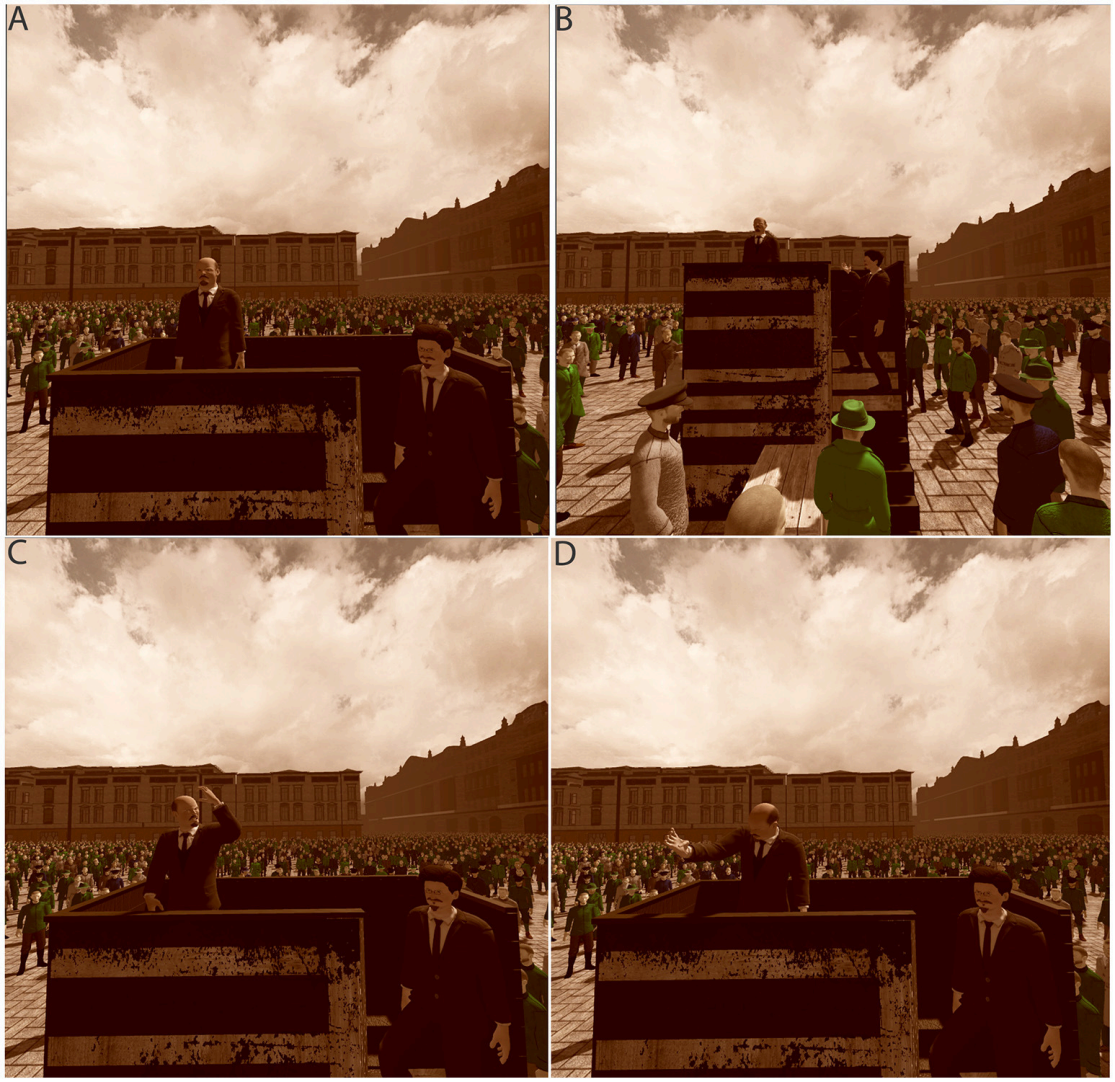
Views from the crowd Sverdlov Square Moscow **(A)** Lenin waiting before starting the speech **(B)** Trotksy signals to start the speech **(C)** and **(D)** Lenin giving the speech. The scene was rendered with a sepia color to make it seem old. The images here were recorded by screen capture, and have been slightly brightened, since in the HMD they look brighter than the screen capture.

The event we chose was based on a famous photograph of Lenin, leader of the Soviet government, giving a speech to Red Army recruits in Sverdlov Square in Moscow in 1920 (tinyurl.com/ya2ymdjk). Leon Trotsky, leader of the Red Army, was standing by the side of the platform. This photograph became notorious, since Stalin later had Trotsky airbrushed out of it. A view of the constructed virtual scene is shown in Figure 1.

Our goal was to bring this photograph to life in immersive virtual reality, where participants could experience being in the crowd, or delivering the speech as Lenin, standing in the place of Trotsky, or even floating above the crowd to obtain a bird's eye view of the scenario. Rather than seeing this as an example of historical cultural heritage, we approached it more as an exercise in “immersive journalism”: “…the production of news in a form in which people can gain first-person experiences of the events or situation described in news stories” (De La Peña et al., 2010). Unlike most applications of “immersive journalism” where participants are mostly onlookers, we designed the virtual reality scenario so that participants would themselves be part of the story.

Virtual reality delivered by a head-tracked head-mounted display (HMD) can be programmed so that the participant's body is apparently substituted by a life-sized virtual body. Hence, when participants look down toward their body, they see the virtual body instead of their real one. Moreover, real-time motion capture can be used so that when the participants move their own body, they see their virtual body move synchronously and in correspondence with their real body movements. As well as seeing the virtual body when they look down toward themselves a virtual mirror can be programmed so that they see a reflection of their virtual body in the mirror. We refer to this as the process of “embodiment” in a virtual body. Such embodiment, which involves seeing the body from first person perspective (1PP) coincident in space with the real body, and synchrony between real and virtual body movements, thus matching proprioception with corresponding visual feedback, typically leads to the illusion of body ownership over the virtual body. The illusion of body ownership is normally associated with synchronous multisensory input that provides evidence about the current state and deployment of the body. The classic example is the rubber hand illusion where subjects experience proprioceptive drift and ownership toward a visible rubber hand, seen in an anatomically plausible position from 1PP and that is seen to be tapped and stroked in synchrony with corresponding tactile input on their corresponding out-of-sight real hand (Botvinick and Cohen, 1998). We have used visuomotor synchrony rather than visuotactile synchrony, together with 1PP, to generate the illusion of ownership over the virtual body (Kokkinara and Slater, 2014). Participants usually have the perceptual illusion that the virtual body is their own, even though of course they know that this is only an illusion.

Moreover, there is growing evidence to support the notion that the type of body itself can have an influence over participants' perception, attitudes, behaviors, and cognition. Thus embodiment of adults in a virtual child body leads to their overestimating object size much greater than embodiment in an adult shaped body of the same height as the child (Banakou et al., 2013; Tajadura-Jiménez et al., 2017). Embodiment of White people in a Black virtual body leads to a reduction in their implicit bias against Black people (Peck et al., 2013; Maister et al., 2015; Banakou et al., 2016) and also to greater mimicry of gestures of postures (a sign of social harmony) (Hasler et al., 2017).

In the experiment described in this paper we had a particular interest in whether embodiment as one of the characters would influence the illusion of presence in the virtual scenario, and the extent to which participants would show signs of enhanced interest in the associated history. Presence refers to the illusion of being in the place depicted in the scenario (Place Illusion, PI) and also the extent to which participants have the illusion that the depicted events are really happening (Plausibility, Psi) (Slater, 2009). Previous evidence suggests that embodiment enhances both PI and Psi (Slater et al., 2010), and this was tested in our experiment. Additionally we were interested in whether embodiment and presence would enhance the probability of individuals later following-up on the history, by accessing a web page that they had been notified of, within 3 weeks after their experimental session.

Normally the embodiment procedure involves participants spending a few minutes in VR looking directly at their virtual body while moving, and also observing the reflected virtual body in the mirror—(e.g., Banakou and Slater, 2014). Here it was important to try to integrate this process into the storyline itself, rather than just something that would be done at the start of the VR exposure but unrelated to subsequent events. To do this we designed a scene that represented Lenin's office. Participants, in the condition of the experiment that involved embodiment as Lenin, were embodied in a virtual body that depicted Lenin, and saw their virtual body both by looking down at it, and as reflected in a mirror. A representation of Trotsky also in the room spoke to the participant embodied as Lenin, and suggested various body movements as gestures that could be made during the forthcoming speech. Hence participants were instructed by “Trotsky” to move while looking at their body and in the mirror. This embodiment period, now part of the overall story, lasted 130 s. After this, participants were automatically transported into the representation of Sverdlov Square, as Lenin standing on top of the platform with the crowd all around, and as in the photograph with Trotsky standing to the left of Lenin. After a few minutes while embodied as Lenin, a short speech would automatically be made, and the gestures of Lenin were pre-programmed except for head movements that were always those of the actual head movements of the participant. At the end of the speech the crowd cheered, and the scenario faded out. Participants also experienced the scenario embodied as a member of the crowd.

The specific issues to be addressed by the experiment were: First, does being embodied as a main character in a story, in this case Lenin, enhance presence in the scenario and interest in the story? Second, does being a character in the story (in this case being part of the crowd) rather than watching it from the outside enhance presence, and interest in the story? A subsidiary issue was whether levels of body ownership commensurate with results of earlier papers could be achieved without full body motion capture but only tracking the head and two hands and using inverse kinematics to continuously infer the upper body pose.

We carried out an experiment with three conditions as follows, where each participant experienced a sequence of scenes:

### Embodied

(A) In the crowd listening to Lenin. (B) Embodied as Lenin in the office carrying out the movements as instructed by the virtual Trotsky followed by speaking to crowd as Lenin. (C) In the crowd listening to Lenin.

### Included

(A) In the crowd listening to Lenin. (B) Watching Lenin in the study carrying out the instructions of Trotsky followed by speaking to the crowd as Lenin. (C) In the crowd listening to Lenin.

### Observing

(A) Floating above crowd listening to Lenin (B) Watching Lenin in the study carrying out the instructions of Trotsky followed by being in the position of Trotsky in the Square listening to Lenin. (C) Floating above crowd listening to Lenin.

Hence in the Embodied condition, participants went through the embodiment process for Lenin's virtual body, and also were in the Square embodied as a person in the crowd. The Included condition was the same except that there was no process of embodiment as Lenin while in the office. In the Observing condition participants never experienced any extended embodiment process, nor were they ever in the crowd itself but only floating above it. They also saw Lenin's speech from the perspective of Trotsky's position on the platform.

The difference between the Embodied and Included conditions was designed to test whether embodiment as Lenin would enhance presence and interest. The difference between the Included and Observing conditions was designed to test whether being part of the story (specifically in the crowd but without the prior embodiment procedure as Lenin) would enhance presence and interest beyond only observing the events—albeit once from the privileged position of Trotsky by the platform. The Observing condition reflects the majority of practice in immersive journalism, where people are only (invisible) observers and not part of the action.

## Methods

### Experimental design

This was a between-groups experiment with a single factor with three levels: Observing, Included and Embodied, as described above. The most important aspect of the three conditions are the differences between them. Hence between the Embodied and Included conditions the difference is that of the embodiment as Lenin in the office. The purpose was to see whether that particular embodiment made a difference to our response variables. The difference between the Included and the Observing condition was that in the latter they were never in the crowd, but always floating above it. Another difference is that instead of speaking as Lenin they were put in the position of Trotsky to hear the speech. The reason for this was to make it as close as possible to the Included condition (i.e., seeing the crowd from the position of the platform) without ever having been embodied as Lenin. So the major difference is never being in the crowd. Regarding the Embodied and Included conditions our primary focus was on the effect of the substantial embodiment as Lenin while in the Office. Regarding the Included and Observing conditions, the primary focus of interest was inclusion as a member of the crowd or not.

Sixty participants were recruited by email around the UCL campus and 20 arbitrarily assigned to each of the three conditions. The mean ± SD age was 24.9 ± 3.34 and 33 of the 60 were males. There were 12 undergraduate students, 24 Masters students, 19 PhD students, 4 research staff, and 1 administrator. Forty out of the 60 had no or little prior experience of VR, with only 1 with substantial experience. The distributions were almost identical across the three experimental groups. Raw data is available in Supplementary Data (some possibly identifying variables have been removed to ensure anonymity of the participants).

The experiment was approved by the UCL Research Ethics Committee, and participants gave written informed consent. They were paid £7 (GB) for their participation.

### The scenario

There were two distinct scenarios and for each one several methods of display.

### Lenin's office

**Figure 2:**
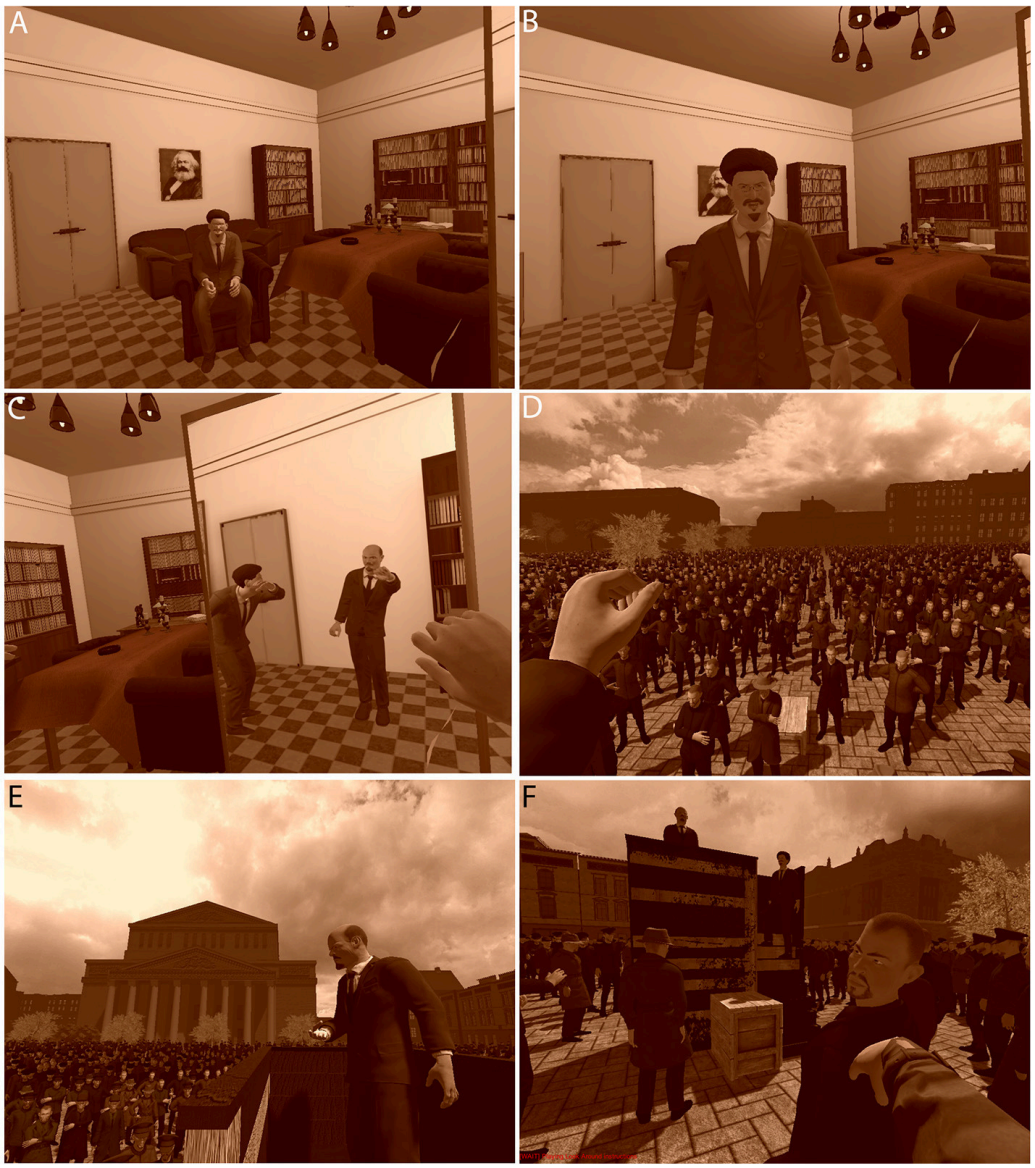
Views of the scenario **(A)** Embodied as Lenin in his office, looking toward Trotsky about to stand up **(B)** Trotsky approaches to stand to the participant's left **(C)** Trotsky is showing the participant gestures that they might make during the later speech as seen in the mirror **(D)** Embodied as Lenin speaking to the crowd **(E)** The view of Lenin giving the speech from the perspective of Trotsky **(F)** Viewing Lenin from the crowd—another crowd member turns to look toward the participant.

There was a reconstruction of Lenin's actual office at the Kremlin, which included the desk, a portrait on the wall of Karl Marx, a Soviet Union map, some bookshelves, and a central table with some couches for visitors (Figures 2A,B). Lenin's virtual body was standing in front of a mirror, and the virtual Trotsky was sitting to his left (Figure 2C). After a while Trotsky started talking explaining that the speech to be given was important, and then he stood up and walked to the left side of Lenin (Figure 2B), and gave various instructions about body movements, such as “Point to the left,” “Point to the right,” and so on (Figure 2C).

This scenario could be experienced by participants in two different ways. In the Embodied condition participants were embodied as Lenin, and the movements of the Lenin body corresponded with the movements of the participant. In the Included and Observing condition participants witnessed a pre-recorded embodiment phase from a third person perspective, located behind and to the left of the Lenin body.

### Sverdlov square

This was a reconstruction of the original photograph (tinyurl.com/ya2ymdjk) and is shown in Figure 1. It depicted the square with 40,191 (male) virtual humans (apart from Lenin and Trotsky) representing Red Army recruits. Lenin was on the platform with Trotsky by the side. After participants had looked around for 30 s, Lenin gave a speech lasting 24 s, which was followed by cheering from the crowd.

This could be experienced by participants in several ways. In the Embodied and Included conditions participants would experience the scenario first in the crowd, then in Lenin's office (either as Lenin or watching Lenin), and then in the crowd again. While in the crowd they would have a virtual body, and the characters standing near to them would react by looking toward them if they happened to touch or collide in any way with those characters. While as Lenin when giving the speech, although gaze direction was always based on the head movements and orientation of the participant (to avoid simulator sickness), all the other actions of the Lenin body were pre-scripted animations. In the Observing condition participants first experienced the scenario floating (4.5 m) above the crowd, then later in the position of Trotsky, and finally again floating above the crowd. While embodied as Trotsky if they would have looked down they would have seen the Trotsky virtual body.

The scenario is illustrated in Supplementary Video 1.

### Procedures

The experiment was carried out in the VR Lab at UCL. Upon arrival at the laboratory participants were assigned a unique ID number used throughout for purposes of anonymity, given information sheets about the study and about the Russian revolution to read, and then asked to read and sign the consent form. They then gave written answers to a demographic questionnaire which provided information on their age, occupation, previous VR experience, and experience with computer games.

They were then introduced to the VR equipment consisting of the head-mounted display (HMD) and hand-held trackers (see below). The procedures were explained to them, and they were asked if they had any questions. They were encouraged to ask questions throughout the setup phase.

They were told where to stand, and also that they should only move their head and upper body, and should not move their feet at all (since their feet were not tracked). Then participants donned the HMD and the four scenarios played out according to which condition they were assigned.

At the end of each distinct scenario (e.g., being in the crowd, being in the office) they were verbally asked a number of questions relating to presence and body ownership, and their answers recorded. During this time of questioning they were still wearing the VR equipment, but the HMD screens were blank.

At the end of all scenarios they removed the HMD and were asked to complete a questionnaire that included questions on their interest in the events. They were also asked to write comments on their experience if they wished.

Finally, they were given a sheet of paper that had a web site address where they could follow up further information about the 1917 Russian Revolutions. They were debriefed about the experiment and then paid.

### Preserving a consistent narrative

Although the primary goal of the experiment was to examine the potential impact of embodiment and inclusion on presence and later follow-up of the history, we wanted to do this in the context of a consistent narrative. Hence, as noted above, the embodiment as Lenin was as part of the story (Trotsky giving Lenin advice on various gestures that should be made during the subsequent speech).

Participants experienced the scenario as part of (or above) the crowd twice—once at the beginning and again at the end, with the Office scene in between. This was because we wanted to give the participants knowledge of what the whole situation was about (hence the first exposure in the crowd) so that when they were embodied as Lenin in the office, they would understand the context, and understand why Trotsky was preparing them to give a speech. Then having had the experience of “being Lenin” both in the office and then on the platform in front of the crowd, they could reexperience being in the crowd.

When participants were embodied as Lenin in the speech scene they had full control over head movements. We decided to leave the Lenin gestures as they had been seen from the point of view of the crowd earlier on, for the purposes of a consistent narrative. Hence, apart from head movements they did not have agency over the movements of the Lenin virtual body while embodied as Lenin during the speech scene. We reasoned that since in that scene there was no mirror, and that participants would be concentrating on the crowd, they might only see the lack of synchrony between their own movements and the virtual body movements sometimes by chance.

As noted the embodiment as Lenin in the office was both explicit and substantial. However, the process of being embodied as a member of the crowd or as Trotsky observing Lenin's speech, in the Observing condition, was very short and simple: A recorded voice said: “Look around the scene and down toward your own body,” followed by the start of the relevant scene.

### Materials

The VR system used was the HTC VIVE, with the HMD and hand trackers. The HMD has a resolution of 2,160 × 1200 (1,080 × 1,200 per eye), a refresh rate of 90 Hz, a field of view of about 110°and weight 0.47 Kg. The headphones used were the Sennheiser HD 206 headphones. The computer used had an Intel i7 4790k processor, 16 Gb of RAM and a NVidia GeForce GTX 1080 graphics card.

### Implementation

The project was implemented within the Unity 3D engine (unity3d.com). The experience is divided into different scenes inside Unity, and in order to be able to retain the participant calibration between all the scenes, we developed a new method providing the ability to change the body representation (the virtual body) many times but calibrating only once. To do this a scene that includes the one master virtual body is maintained, with full body tracking but with all its render components (meshes) turned off. Then in each specific scene opened additively there is a “slave” virtual body that copies all the movements from the master one, and moves the virtual camera to the head of the slave.

To recreate the crowd in the Sverdlov Square scene we used high polygon meshes for close up characters, while per-joint impostors (Beacco et al., 2012) were used for distant ones. These had to be implemented for Unity, and combined with instancing and palette skinning techniques (Dudash, 2007) in order to obtain real-time framerates suited for VR.

Specific characters such as Lenin were initially modeled using Adobe's Fuse software for sketch versions, and iteratively refined by our artist using other 3D modeling software such as Autodesk's Mudbox and Maya. Maya was also used to model most of the 3D assets in the scenes, such as buildings and props.

The sound of Lenin's speech was taken from https://www.youtube.com/watch?v=nap0u7QEAPU&feature=youtu.be%27%27. This shows an actor playing the role of Lenin. The original scene could not have been recorded in 1920 since the technology for film with sound was not yet available.

This was implemented using the 3D audio settings of Unity^1^ with the spatialBlend set to 1.0. While participants in the Embodied and Included conditions were embodied as Lenin giving the speech they would have heard the Lenin voice as in-the-head stereo. This was the same method used by Banakou and Slater (2014) who showed that participants in an embodied condition would also have illusory agency over the speaking of their embodied character. It was not the goal of this experiment to study this aspect, however.

### Response variables

There are several types of response: presence (Place Illusion and Plausibility), body ownership and agency with respect to the crowd member and Lenin body, the intention to follow-up, and the degree of curiosity and interest in the historical events. The last three we refer to collectively as Engagement. Each of these were assessed on multiple questions.

A further response variable (*website*) records whether or not participants followed-up by accessing the web site address that had further information about the Russian Revolution. This is therefore a binary variable, 0 meaning that they did not access the web site and 1 meaning that they did.

### Statistical methods

The results are presented in two forms. First we consider the raw data, showing what actually happened with this sample, in terms of presence, body ownership, engagement, and follow-up. These results are presented in the form of box plots and bar charts with corresponding commentary.

Second we carry out formal analysis to examine the hypotheses presented in the introduction. Polychoric principle components analysis is used to combine several questionnaire variables relating to one concept (e.g., Place Illusion) into one variable on a continuous scale. Polychoric PCA (Olsson, 1979) treats ordinal variables as arising from cutoff points on a continuous underlying latent variable with a Normal distribution. It thus avoids treating ordinal responses as continuous. For this purpose we used the function “polychoricpca” (Kolenikov and Ángeles, 2004) in Stata 15 (https://www.stata.com).

For the formal analysis we use a Bayesian approach, since this enables the simultaneous evaluation of several equations corresponding to the hypotheses in one overall model. The model is introduced below after its variables have been specified. For the Bayesian analysis we used the Stan system (Carpenter et al., 2016) (http://mc-stan.org).

## Results

### Presence in Sverdlov square

**Figure 3:**
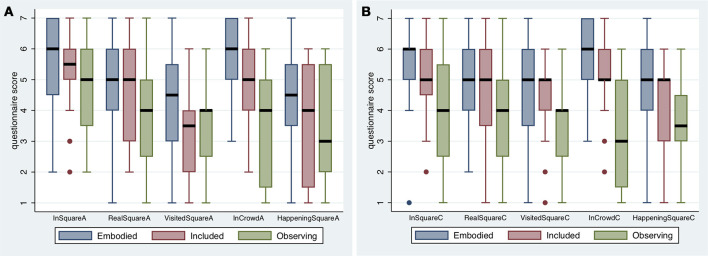
Box plots results on the questions relating to presence in periods **(A**,**B)**. The thick horizontal lines are the medians, and the boxes the interquartile ranges (IQR). The whiskers extend from max(min value, 25%tile-−1.5*IQR) to min(max value, 75%tile + 1.5*IQR).

Participants were asked a series of questions relating to Place Illusion (PI) and Plausibility (Psi) immediately after they had experienced being in Sverdlov Square in periods A and C of the experimental design (Table 1). Figure 3 shows the resulting box plots.

**Table 1:** The questionnaires: each statement was assessed on a 1–7 Likert Scale.

**Variable**	**Question**	**Scale**	**Administered**
**PLACE ILLUSION**			
InSquare	I had the sensation of being in the square amongst the crowd listening to Lenin's speech.	1: Not at all 7: Very much so	After exposures A and C in all experimental conditions
RealSquare	There were moments when the square seemed more real to me than the lab.	1: The Lab 7: The Square	After exposures A and C in all experimental conditions
Visited	When I think back on the events the memory seems more like images I saw rather than somewhere I visited.	1: Images 7: Visited	After exposures A and C in all experimental conditions
InCrowd	I had the sensation of being in with a crowd of people.	1: Not at all 7: Very much so	After exposures A and C in all experimental conditions
**PLAUSIBILITY**			
Happening	I had the sensation that the events taking place in the virtual world were really happening.	1: Not at all 7: Very much so	After exposures A and C in all experimental conditions
**BODY OWNERSHIP AND AGENCY**			
DownCrowd	Although the body I saw when I looked down toward myself did not look like me, I had the sensation that it was my body.	1: Not at all, 7: Very much so	After exposures A and C in the Embodied and Included conditions
MyBodyCrowd	My overall feeling was that although the body I saw from a first-person perspective did not look like me, I had the sensation that it was my body.	1: Not at all, 7: Very much so	After exposures A and C in the Embodied and Included Conditions
AgencyCrowd	The body I saw from a first-person perspective moved in accordance with my movements.	1: Not at all, 7: Very much so	After exposures A and C in the Embodied and Included Conditions
MirrorLenin	Although the body that I saw in the mirror did not look like me I had the sensation that it was my body.	1: Not at all 7: Very much so	After exposure B in the Embodied Condition
DownLenin	Although the body I saw when I looked down toward myself did not look like me, I had the sensation that it was my body.	1: Not at all, 7: Very much so	After exposure B in the Embodied and Included Condition
MyBodyLenin	My overall feeling was that although the body I saw from a first-person perspective did not look like me, I had the sensation that it was my body.	1: Not at all, 7: Very much so	After exposure B in the Embodied and Included Condition
AgencyLenin	The body I saw from a first-person perspective moved in accordance with my movements.	1: Not at all, 7: Very much so	After exposure B in the Embodied Condition
**ENGAGEMENT**			
Interested	I have become more interested than before in finding out about the 1917 Russian Revolution.	1: Not at all, 7: Very much so	After the VR session in all Conditions
Followup	I am likely to follow up to try to discover more about the particular event depicted.	1: Not at all, 7: Very much so	After the VR session in all Conditions
Curiosity	Being in the scenario seemed to me to be a way to heighten my curiosity about these events.	1: Not at all, 7: Very much so	After the VR session in all Conditions

*The questions were administered after exposures A and C*.

The most striking feature is that for all cases the medians for Observing are less than for Embodied, and for all but one case (VisitedSquareA, where the difference is negligible) the median for Observing is less than for Included. For this sample of participants, the Observing levels of PI were on the whole less than the other conditions.

Polychoric PCA was applied to the scores for *InSquare, RealSquare, VisitedSquare, InCrowd* for both A and C together, to construct one overall continuous scale measure of Place Illusion. The first principle component explained 61% of the overall variation, and the second principle component 14%. We refer to the variable corresponding to the first component *ypi*. The variable corresponding to the second component was found to have no relationship with any other variable, and is not considered further. The variable *ypi* is positively correlated with each of the questionnaire scores with each value of Spearman's ρ > 0.54 (*n* = 60).

Polychoric PCA was also applied to the scores *HappeningSquareA* and *HappeningSquareB*, to produce a new variable *ypsi*, which accounts for 84% of the total variance. This variable represents the overall level of Plausibility. Both Spearman's ρ > 0.88.

The variables representing PI (*ypi*) and PSI (*ypsi*) are highly correlated, with *r* = 0.82 (*n* = 60). Hence due to multicollinearity, they cannot be used simultaneously as independent variables in analysis, therefore Polychoric PCA was applied to all of the questionnaire scores in Figure 3, to produce one overall measure of presence (*ypres*), which explains 62% of the total variance. All Spearman's ρ > 0.59 (*n* = 60) with respect to the original scores.

**Figure 4:**
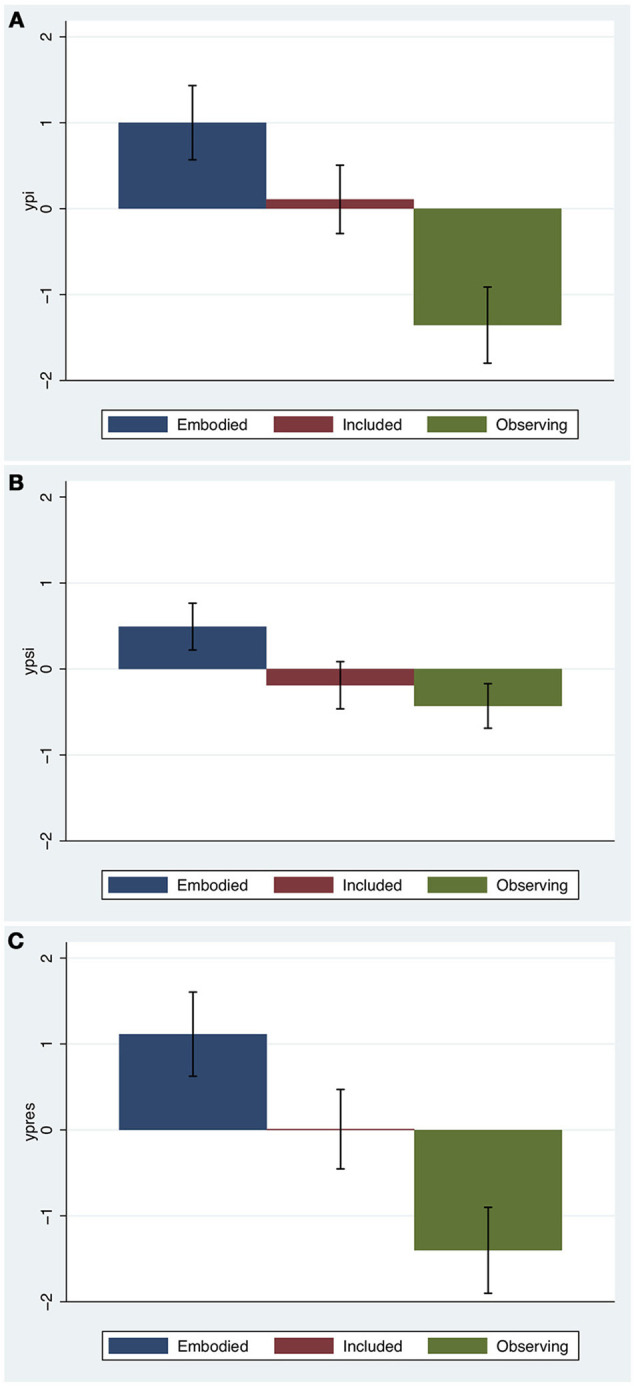
Bar charts for the presence composite variables by condition. The heights show the means and the whiskers the standard errors. **(A)** Place Illusion, **(B)** Plausibility Illusion, **(C)** Overall presence.

Figure 4 shows the means and standard errors for the new variables, by condition. For this sample, Place Illusion (*ypi*) is clearly highest for the Embodied group and lowest for the Observing group. In the case of Plausibility (*ypsi*) the Embodied group has the highest scores, but there is little difference between the Included and Observing groups. For overall presence (*ypres*) the Embodied group has the highest score and the Observing group the lowest by far. (Formal analysis is presented later).

### Body ownership and agency

The questions regarding body ownership and agency were not administered to all participants. Regarding the crowd scene these questions were appropriate for those in the Embodied and Included groups after exposures A and C, and those relating specifically to the embodiment as Lenin only to those in the Embodied group after exposure B. The purpose of these questions was to examine the extent to which the conditions influenced the illusion of body ownership—with respect to Lenin, and with respect to being a member of the crowd.

**Figure 5:**
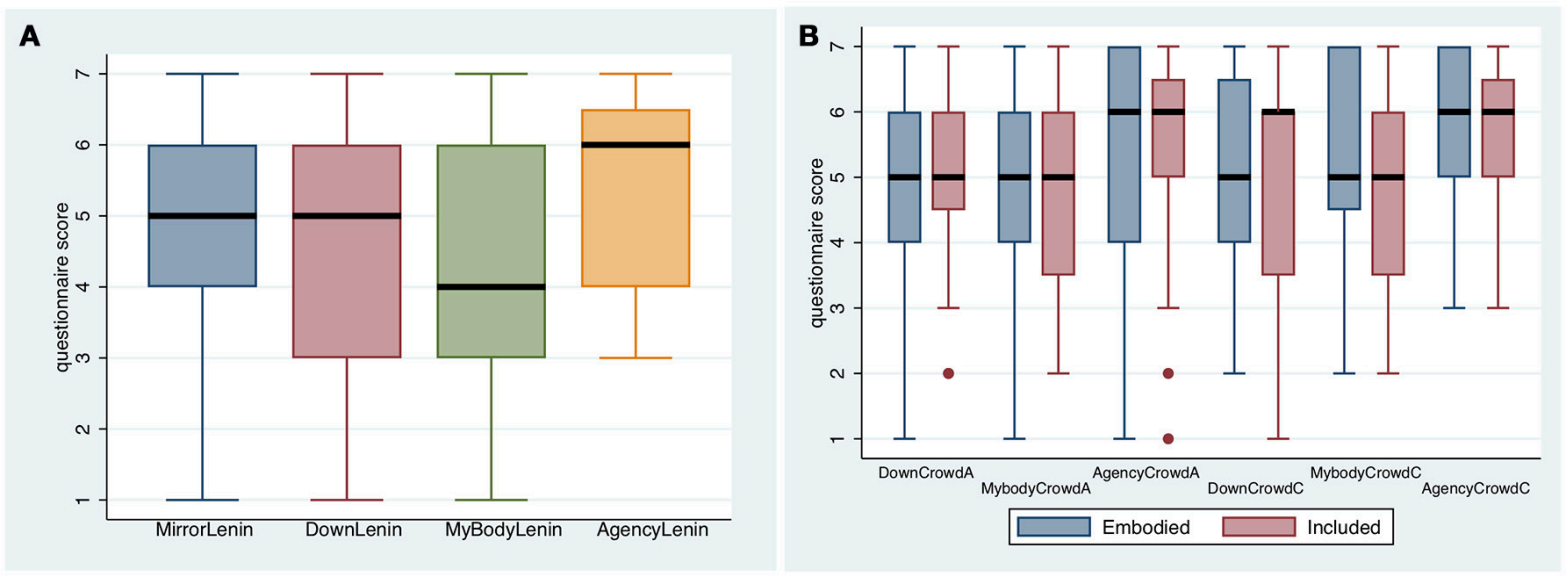
Box plots for questions related to body ownership and agency (Table 1) **(A)** with respect to embodiment as Lenin, and **(B)** with respect to being in the crowd.

Figure 5A shows the results for embodiment as Lenin in the study. All median scores are at least 4, and for the feeling of ownership of the Lenin body as seen in the mirror the lowest quartile is above 4. The median score for the overall feeling of being Lenin (*MyBodyLenin*) is 4, though the distribution is skewed toward the upper tail. *AgencyLenin*, the extent to which the Lenin body moved in accordance with the participant's actual movements, scored highly with a median of 6, and lower quartile of 4. This is interesting because in fact the body movements were reproduced from only three tracking points (head, and two hands) using inverse kinematics.

Figure 5B refers to body ownership and agency with respect to being in the crowd. We can see that there is no difference between the Embodied and Included conditions, all the medians are at least 5, and all the lower quartiles are at or almost 4. A difference between these conditions would not be expected with respect to being in the crowd.

Since it was not sensible to ask these questions across all conditions, these are not included in the formal analysis below, and the results are given here for information about the success of the embodiment procedures.

### Engagement

**Figure 6:**
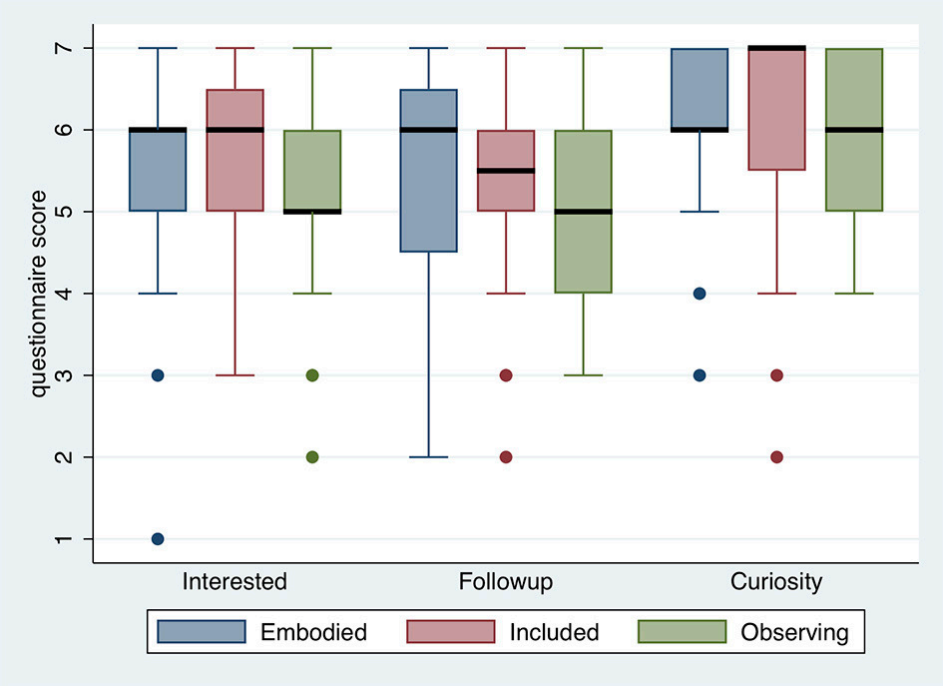
Box plots for the Engagement questions.

Figure 6 suggests that there may be less engagement in the Observing condition, the median being least for this case on two of the three scores, although, there is considerable overlap between the IQRs. Overall the scores are high in all conditions, though with some outliers. Polychoric PCA was used to create the new continuous variable *yengage*, which explains 79% of the total variation. All Spearman's ρ > 0.83 (*n* = 60).

**Figure 7:**
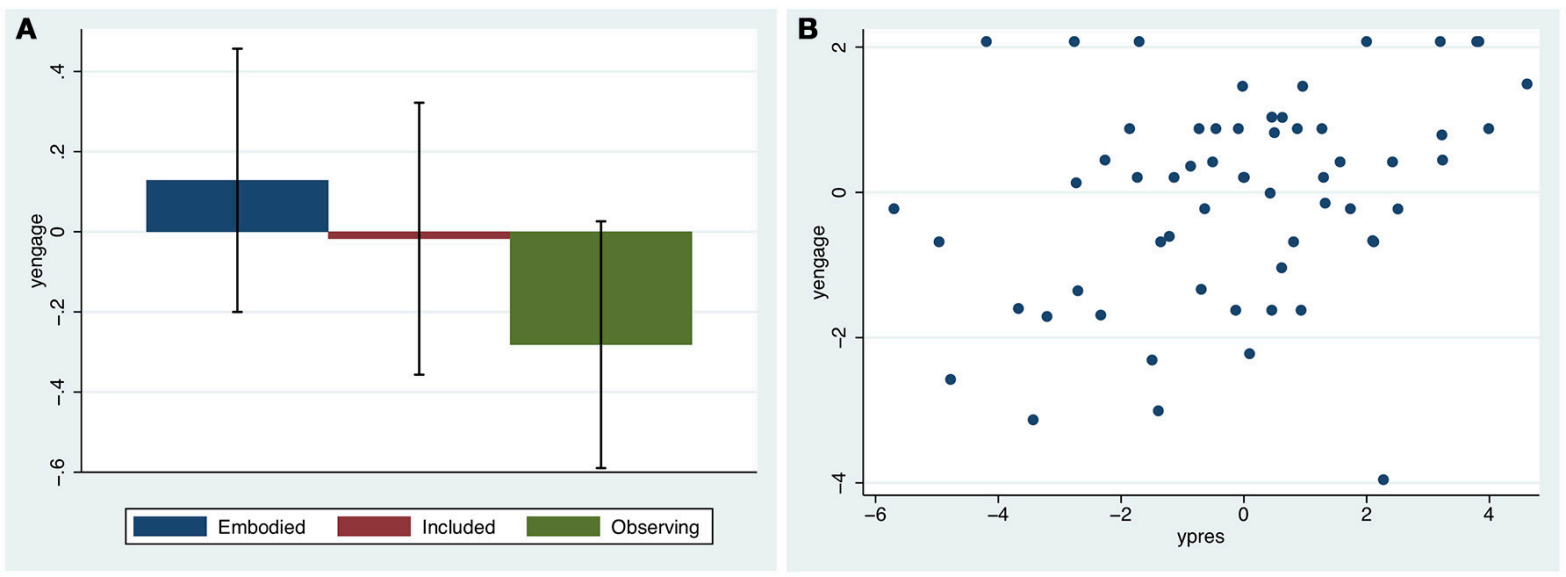
Engagement (*yengage*) **(A)** Bar chart of means and standard errors of yengage by condition **(B)** Scatter diagram of *yengage* by *ypres*.

Figure 7A shows the means and standard errors of *yengage*. While the largest mean is for Embodied, and the smallest for Observing, the standard errors are high showing no clear advantage for the conditions. However, Figure 7B shows a strong linear relationship between Engagement and Presence (*r* = 0.32, *n* = 60) although note the many outlying points.

### Follow-up

Of the 60 participants 10 followed-up by accessing the web site within the 3 week period allowed. All but one did so on the same day that they completed the experiment. Three of the 10 were in the Embodied condition, 5 in the Included and the remaining 2 in the Observing condition. Whether or not the participant followed-up is a binary variable (*website*) with a score of 1 if they accessed the web site and 0 otherwise.

### The bayesian model

The statistical model postulates that PI (*ypi*) and PSI (*ypsi*) are dependent on the experimental conditions (Embodied (*E*), Included (*I*), Observing), Engagement (*yengage*) is dependent on the conditions and the level of presence (*ypres*), and similarly the number of website visits (*website*) is dependent on the conditions and the level of presence. In first fitted models no relationship was found between *yengage* and the conditions, but a strong relationship with presence. On the other hand, *website* showed no relationship with presence, but some relationship with the conditions. The fitted model is as follows:

Likelihood functions:

(1) $$\begin{array}{rcl} y_{pi,i} & \sim & N\left(\beta_{pi,0}+\beta_{pi,1}I_{i}+\beta_{pi,2}E_{i},\sigma_{pi}\right) \end{array}$$

(2) $$\begin{array}{rcl} y_{psi,i} & \sim & N\left(\beta_{psi,0}+\beta_{psi,1}I_{i}+\beta_{psi,2}E_{i},\sigma_{psi}\right) \end{array}$$

(3) $$\begin{array}{rcl} y_{engage,i} & \sim & Student_{t}\left({v,\beta }_{engage,0}+\beta_{engage,1}y_{pres,i},\sigma_{engage}\right) \end{array}$$

(4) $$\begin{array}{rcl} website_{i}\; & \sim & \;bernoulli\left(\frac{1}{1+exp\left\{-\left(\beta_{web,0}+\beta_{web,1}I_{i}+\beta_{web,2}E_{i}\right)\right\}}\right) \end{array}$$

$$\begin{array}{r} i=1,\ldots ,n \end{array}$$

Prior distributions:

All $\beta_{{}^{*}}\;\sim \;N\left(0,10\right)$
All $\sigma_{{}^{*}}\sim Cauchy\left(0,5\right)$
*v* ~ exponential(mean 10).

Equations (1)–(3) give the likelihood functions for the model. The notation *y*~*N*(μ, σ) means that y has a normal distribution with mean μ and standard deviation σ. *y*_*pi, i*_, *y*_*psi, i*_, *y*_*engage, i*_, *website*_*i*_, *y*_*pres, i*_ are the *i*th observations on *ypi, ypsi, yengage, website*, and *ypres*, respectively, with *n* = 60. *I*_*i*_ and *E*_*i*_ are the Included and Embodied levels for the *i*th individual, with the value 1 corresponding to being in that condition, and 0 otherwise. (Observing cannot be also included in the model otherwise the design matrix does not have full rank).

Equations (1) and (2) specify standard normal linear models for *ypi*, and *yspi* respectively, with parameters $\beta_{{}^{*}}$ and $\sigma_{{}^{*}}$.

As we have seen above *yengage* has several outliers and therefore rather than model this by a normal distribution and deleting the outliers, we chose the Student t-distribution, with unknown degrees of freedom υ≥1 and mean the linear model in *ypres* and scale parameter σ_*engage*_. The Student t-distribution has much wider variance, indeed the variance is infinite for ν = 1. As shown in Figures 6, **7A** there is no influence of condition on *yengage*, and an earlier model that included these terms supported that conclusion.

The variable *website* is treated as a Bernoulli random variable with probability given by the inverse logistic function of the linear model in Included and Embodied, following a standard logistic model. An earlier model that included *ypres* as a possible explanatory variable was not supported, and therefore this variable was removed from the final model.

The prior distributions for the $\beta_{{}^{*}}$ are all N(0,10) giving an effective parameter range of approximately −30 to 30, or 95% credible intervals ±19.6. The prior distributions for the standard deviations $\sigma_{{}^{*}}$ are all Cauchy distributions with scale parameter 5, but restricted to have positive support. Note that the Cauchy distribution has infinite variance, but nevertheless is a proper distribution and therefore preferable to using a flat prior.

The prior distribution of the degrees of freedom of the t-distribution *v* was chosen as exponential with mean 10. Therefore the standard deviation is also 10, which gives a large range of effective possible values for *v*, including the possibility that yengage might be modeled by a Normal distribution (for *v* ~30 or more).

The hypotheses presented in the introduction translate to $\beta_{{}^{*}}>0$. Hence we are interested in the posterior probabilities $P\left(\beta_{{}^{*}}>0|Data\right)>0$.

The model fit was carried out with the Stan system, using 4,000 iterations. The model converged, with all Rhat = 1. Results are shown in Table 2. Looking particularly at the last column the evidence suggests that both Included and Embodied contribute positively to PI, and in particular Embodied contributes to Psi. Engaged is positively associated with the overall presence (PI and Psi combined). There is some evidence that Included contributes to the probability of accessing the web site subsequent to the experiment.

**Table 2:** Posterior estimates, standard errors and 95% credible intervals and posterior probabilities that the parameters are positive.

**Parameter**	**Coeff. of**	**Mean**	**S.E**.	**2.5%tile**	**Median**	**97.5%tile**	P(β_*_>0 | Data)
**PI**							
β_*pi*, 0_		−1.34	0.007	−2.18	−1.34	−0.49	0.002
β_*pi*, 1_	Included	1.45	0.010	0.29	1.46	2.62	0.992
β_*pi*, 2_	Embodied	2.34	0.010	1.16	2.34	3.54	1.000
σ_*pi*_		1.93	0.003	1.62	1.92	2.34	
**PSI**							
β_*psi*, 0_		−0.43	0.004	−0.96	−0.43	0.10	0.064
β_*psi*, 1_	Included	0.24	0.006	−0.50	0.24	0.99	0.730
β_*psi*, 2_	Embodied	0.92	0.006	0.17	0.92	1.66	0.990
σ_*psi*_		1.22	0.002	1.02	1.21	1.48	
**ENGAGED**							
β_*engage*, 0_		0.01	0.003	−0.35	0.01	0.37	0.517
β_*engage*, 1_	*ypres*	0.22	0.001	0.07	0.22	0.37	0.998
σ_*engage*_		1.25	0.002	0.96	1.25	1.59	
ν		13.43	0.153	3.13	10.73	38.96	1.000
**WEBSITE**							
β_*web*, 0_		−2.39	0.013	−4.22	−2.32	−1.05	0.000
β_*web*, 1_	Included	1.21	0.015	−0.57	1.16	3.27	0.915
β_*web*, 2_	Embodied	0.53	0.016	−1.48	0.51	2.63	0.689

*The prior 95% credible intervals for the β parameters are all −19.6 to 19.6. The prior distributions for the σ parameters have infinite variance*.

### Goodness of fit

In order to assess goodness of fit, the Stan functions generated 4,000 pseudo random observations using the fitted model on each of the response variables *y*_*pi, i*_, *y*_*psi, i*_, *y*_*engage, i*_, *i* = 1, …*n*. The 95% credible intervals were computed for *each individual* (*i*) for each response variable and compared with the actual observation.

In the case of *website*, instead the estimated probabilities were computed using the means shown in column 3 of Table 2. The estimated probabilities are

$$\begin{array}{l} {\hat{p}}_{i}=\;\frac{1}{1+exp\left\{-\left({\hat{\beta }}_{web,0}+{\hat{\beta }}_{web,1}I_{i}+{\hat{\beta }}_{web,2}E_{i}\right)\right\}} \end{array}$$

where the ${\hat{\beta }}_{web{,}^{*}}$ are the means of the posterior distributions.

**Figure 8:**
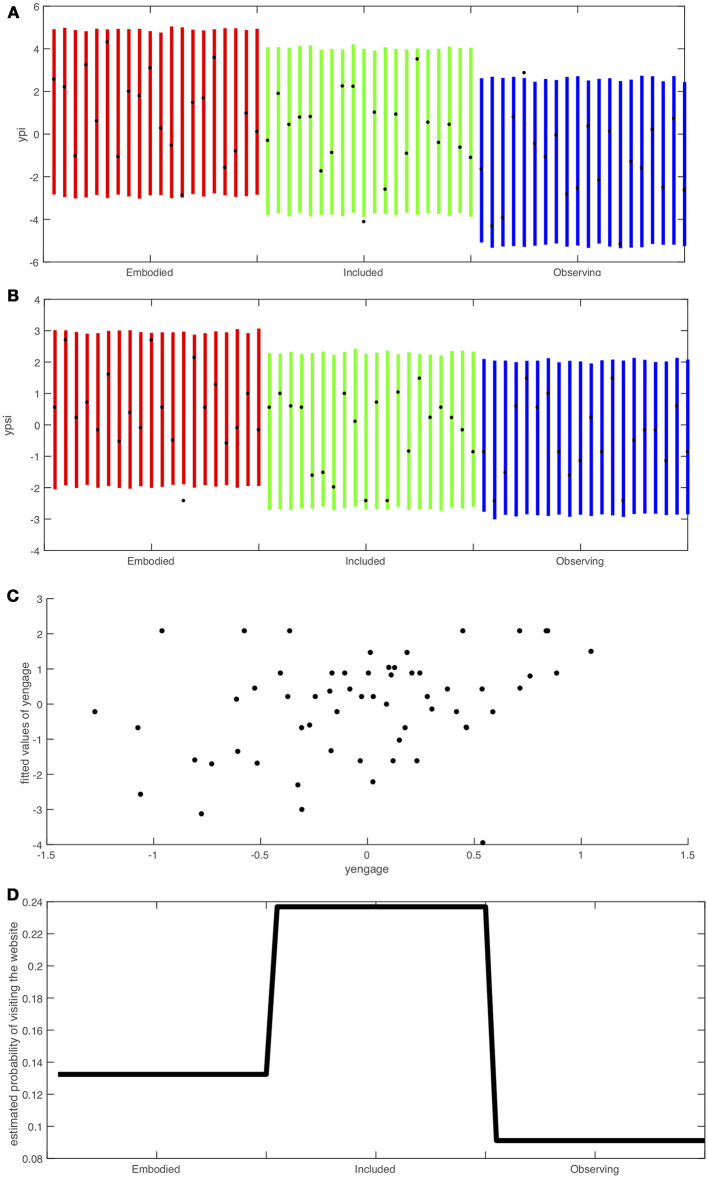
Credible intervals and fitted values of the model corresponding to Equations (1)–(4) and Table 2. **(A)** Credible intervals for the ypi scores of each individual with the black dots the observed values, separated into Embodied, Included and Observing conditions. **(B)** Credible intervals for the ypsi scores of each individual with the black dots the observed values, separated into Embodied, Included and Observing conditions. **(C)** Fitted values of yengage by observed values. **(D)** Probabilities of the website follow-up by Embodied, Included, and Observing conditions.

The goodness of fit results are shown in Figure 8. It is important to note that Figures 8A,**B** are credible intervals for the *individuals* rather than means. These graphs lend support to the model since almost all of the observed values fall within the credible intervals. Moreover the pattern of credible intervals for the individuals match the findings of the overall model: for *ypi* the intervals for the Embodied group are higher than for the Included group which are higher than for the Observing group. Similarly for Figure 8B (*ypsi*) only the Embodied group is higher. Figure 8C shows a reasonably good correspondence between the observed and predicted values for *yengage*. Finally the estimated probabilities in Figure 8D show, corresponding to the observations, that the Observing group would be predicted to be least likely to followup.

## Discussion

This study examined the impact of three different types of relationship between a participant and a historical event depicted in virtual reality. Participants were either embodied as the main protagonist (Lenin) preparing to give a speech, were embodied in Lenin giving the speech, and also experienced the scenario as part of the crowd to whom the speech was addressed (Embodied). Or, they saw the speech as part of the crowd (Included), or they had the role of observers floating above the crowd (Observing). The results show that the Embodied and Included conditions contribute far more to the Place Illusion component of presence than the Observing, but with respect to Plausibility only Embodiment clearly has the greatest contribution. Overall the illusion of presence is positively associated with the degree of engagement (interest, curiosity, follow-up). Participants in the Included condition were more likely to actually follow up by linking to the web page than in the Observing condition. Participants in the Observing condition were less likely to follow-up than in the other two conditions.

The major difference between the Embodied and Included conditions is that there is an explicit embodiment process as Lenin, in the Embodied condition. In both conditions there is also embodiment as a member of crowd. The results of this experiment suggest that the explicit embodiment process as Lenin does contribute more to Plausibility (i.e., the illusion that the events are really happening). The recommendation is that if the VR setup permits this (i.e., real-time motion capture), it is worth including an explicit embodiment process, and otherwise embed and embody participants in the scenario as much as possible. Avoid participants simply being disembodied observers, since this seems to result in the least satisfactory outcome.

Normally the embodiment process is carried out separately from the main experience. For example in Osimo et al. (2015) participants first had a period of embodiment in a virtual Sigmund Freud body by being asked to move while observing their virtual body from 1PP directly and also in a virtual mirror, and then after that the main scenario started. In the case of this study we have made the Lenin embodiment as part of the story itself. Of course it would have been extremely unlikely that Leon Trotsky would have been instructing Lenin in this way in reality, but here we used a dramatic means to integrate the embodiment process as a natural part of the story-line, without taking anything away from the main event (the speech to the crowd itself).

An important interest we had in the design of this experiment is whether the simplified embodiment process, involving only head and two hand tracking (making use of the VIVE HMD and the two hand-held wands) would be sufficient to induce the illusion of body ownership. We can compare the results with the closest other experiment that we have carried out, where participants were also embodied in an older male, representing Sigmund Freud (Osimo et al., 2015). In that experiment participants were tracked in real-time using a whole body, highly accurate motion capture suit. Participants were embodied in a virtual body that was a copy of their own, or as Freud. Figure 2 of that paper^2^ shows two questions corresponding to *MirrorLenin, DownLenin*, and *DownCrowd* (see the results for Sync and Freud). In the “Freud” embodiment, the “Down” and “Mirror” questions both had a median of 6 with small interquartile ranges, and the equivalent of the “Agency” question (MyMovements) had a median of 7. The comparable medians for the Lenin embodiment are one point lower, but more importantly the interquartile ranges are greater. Similarly for embodiment as a member of the crowd. Of course the experimental scenarios were quite different, although both had an explicit period of embodiment (as Freud, as Lenin). Bearing in mind that it is not appropriate to do a formal statistical analysis of data from these two different experiments, the results do suggest that the level of body ownership and agency is likely to be lower in the type of setup we used in this experiment with only head and two hands actually tracked, compared to the full body tracking of the previous experiment. In particular during the Lenin embodiment the feet were not tracked at all, so that if participants happened to move their feet or take a small step, then the representation of Lenin would become distorted (for example, if the participants stepped forward then the Lenin body would be hunched forward).

The results are nevertheless encouraging. In spite of the fact that the body tracking was less accurate than we normally employ, and also that while giving the speech to the crowd embodiment of Lenin did not include real-time motion capture (apart from the head) so that there would have been asynchrony between actual and virtual body movements, the body ownership scores were high, and the agency score was very high (a median of 6 out of a possible maximum of 7). This could be for two reasons: (i) as mentioned above the embodiment as Lenin in the office was substantial. (ii) It is likely that almost all the time the participant would not have noticed the lack of control over the Lenin body while giving the speech—since unlike in the office there was no mirror and participants would be focusing on the crowd. Participants may have noticed the lack of synchrony by chance when e.g., Lenin's animated arms came into view without the participant's own arms having made that gesture. Anecdotally we observed that some participants attempted to match the movements of the Lenin virtual body when they saw these.

In more recent work the problem of lack of movement synchrony has been further addressed with the deployment of three extra VIVE trackers, two for the feet, and one for the trunk, which overall produces virtual body movements even closer to the actual movements.

The number of follow-ups, that is people who later accessed the web page does not in itself seem high. Nevertheless 8/40 in the Embodied and Included conditions did so (20%). Tim Fiennes, the Head of Audience Research for Emerging Technology and Distribution at the BBC, informed the authors (Personal Communication) that “The findings from the Being Lenin research are promising and indicate the huge potential VR has to create memorable experiences. In particular the experience of ‘being embodied’ suggests users are more likely to find out more on the topic compared to what we'd expect for traditional media. Further work is required to see if this can be achieved at scale.”

The approach to history is related to two issues of interest in the application of virtual reality. The first is the use of teaching of history using VR. Allison (2008) argued that VR would be useful for history education for three main reasons: First, historical events can be depicted in several different ways, showing interpretations from different points of view. We would add that as well as depicting events in many different ways, even depicting them in one way but allowing learners to inhabit different characters in the story, and understand the events from these different points of view, could be useful. In the experiment described here we had two main points of view—that of the protagonist (Lenin) giving the speech, and that of a crowd member listening to the speech. We found that the level of presence was quite different in those cases compared to mainly observing the scenario from a disembodied point of view. Second, Allison (2008) argues that VR could lead to a quite different approach to the teaching of history. However, he notes that virtual worlds suffer from a lack of solidity, that reaching out to touch something and feeling nothing, lessens the illusion, which is true. It was argued in Slater (2014) that the lack of generalized haptics in VR is one of the major challenges in this field. Third Allison (2008) points out that there are vigorous debates about what is “the past” and correspondingly what is the “real” history. VR is excellent for this type of debate, since we can show how experiencing the same event from different points of view can lead to different understandings of the event. Although this was not the goal of our experiment it is clear that the type of approach we have adopted, embodying participants in the scenario itself as one of the characters, could be used for this purpose.

More recently Ijaz et al. (2017) used Second Life to create an ancient city that gave students the ability to interact with virtual agents representing its inhabitants. Three groups of 20 students were recruited—one that used traditional methods of learning (reading etc.), the second saw a video about the city, and the third experienced the city in Second Life. Note that Second Life as used in that study was non-immersive, since participants interacted on a screen rather than HMD. The study found that those in the Second Life group later outperformed those in the other two groups on a questionnaire that tested their acquired knowledge. It should be noted that there was no control of the time that participants could spend on the method to which they were assigned, since they could engage in the learning activity for as long as they wanted. Hence the results could be due to time differences. However, it is encouraging that the method that required greater interaction, by allowing participants to navigate through the virtual city and converse with the virtual inhabitants, did result in the best performance—and if this is mainly because they spent more time with this system, then that is anyway a useful finding.

A second major interest in the application of VR is to the reporting of news stories. This is generally referred to as “immersive journalism.” Although the event that we considered took place nearly 100 years ago what we have learned applies equally well to immersive journalism. What is different, of course, is how materials are sourced—we cannot interview the protagonists, for example, and can only rely on archive material in the case of reconstruction of historical events.

The first immersive journalism production (De La Peña et al., 2010), gave participants an experience of being in a cell waiting to be interrogated, based on documentation about the interrogation of a Guantanamo Bay prisoner. The participant was embodied with real-time feedback of body movements, and even though they were comfortably seated, their body, seen from their own first person perspective by looking down toward themselves, and in a virtual mirror, was depicted as standing in a stress position. This led to participants feeling discomfort and some anxiety. However, subsequent immersive journalism pieces have typically provided scenarios that are experienced passively, with no or very little interaction possible by the user. For example, Emblematic's (the company of de la Peña) “Hunger in Los Angeles” (Sundance Film Festival, 2012) depicted events on a food bank queue, where one of the (virtual) people was seen to collapse due to diabetes. Emblematic's “Project Syria” (2014 World Economic Forum) was about a bombing of civilians in the Syrian civil war. Their “One Dark Night” and “Kiya” were respectively about the shooting of a teenager and a murder resulting from domestic violence. The method of de la Peña, has been to combine data from the original events (such as police recordings) into a 3D graphics constructed scenario. More recently photogrammetry was used to produce highly realistic “After Solitary” which depicts the story of a prisoner who was in solitary confinement for 5 years. In all these examples, the user was invisibly present in the scenario as a passive observer.

The BBC has also experimented with graphics model-based virtual reality, most notably in their piece about the refugee crisis “We Wait.” This depicted a group of refugees waiting on a beach for a boat to arrive to take them to Europe. Participants were embodied in a virtual body. This piece was the subject of a recent experimental study (Steed et al., under review) where some level of virtual character responsiveness (when participants looked at a virtual character the character would return the gaze) was compared with no such responsiveness. The study found, in line with our current results, that the responsiveness led to a dramatic improvement of the illusion of presence over no responsiveness, with some evidence that it also led to a higher chance of follow-up after the experiment.

Much of the other work in this field uses 360 degree video rather than model-based graphics. For example, Louis Jebb's and Edward Miller's company Immersiv.ly produced “Hong Kong Unrest” about the demonstrations in Hong Kong in 2015. Gabo Arora's and Chris Milk's “Clouds over Sidra” is a United Nations sponsored VR documentary film about a child refugee in the Syrian war.

News organizations and broadcasters including The New York Times, The Guardian and the BBC have developed a number of other 360 degree VR news stories or documentaries. For further reviews of immersive journalism see (Doyle et al., 2016; Slater and Sanchez-Vives, 2016; Watson, 2016).

While many immersive journalism pieces have very high production standards, their passivity is a drawback. The point of using VR to depict events is because uniquely VR can provide the powerful illusions of “being there” and that the events depicted are “really happening.” Very early VR experiments showed that embodiment enhances presence (Slater and Usoh, 1993), and both the Place Illusion and Plausibility aspects of presence (Slater et al., 2010).

Our overall conclusion is that in immersive journalism and the depiction of historical events in VR should embody participants (so that when they look down toward themselves they have a visible body), and embed participants in the scenario (they are part of it, not simply observing it).

## Author contributions

MS formulated the original idea, and the design of the study was by MS and ZW. The program was implemented by XN, AB, RO, JV. JV did the modeling of the scenario and characters. The experiment was carried out by JT. The statistical analysis was by MS. MS wrote the paper with contributions from all the authors. All authors revised the manuscript.

### Conflict of interest statement

The “Being Lenin” program was implemented by Virtual Bodyworks and was funded by the BBC, for artistic, demonstration and testing purposes, and this study was carried out subsequent to the original work. There are no financial interests for Virtual Bodyworks or the BBC regarding the results of this study. MS is a Founder of Virtual Bodyworks S.L. The remaining authors declare that the research was conducted in the absence of any commercial or financial relationships that could be construed as a potential conflict of interest.

## Footnotes

^1^https://docs.unity3d.com/ScriptReference/AudioSource-spatialBlend.html

^2^https://www.nature.com/articles/srep13899/figures/2

## Supplementary material

Supplementary Video 1 A video of the main features of the experiment.

Supplementary Data The raw data.
